# Graduate Midwifery Education in Uganda Aiming to Improve Maternal and Newborn Health Outcomes

**DOI:** 10.5334/aogh.2804

**Published:** 2020-05-21

**Authors:** Edward Kumakech, Julie Anathan, Samson Udho, Anna Grace Auma, Irene Atuhaire, Allan G. Nsubuga, Bonaventure Ahaisibwe

**Affiliations:** 1 Department of Nursing and Midwifery, Lira University, UG; 2 Seed Global Health, US

## Abstract

**Background:**

Maternal and newborn health outcomes in Uganda have remained poor. The major challenge affecting the implementation of maternal and newborn interventions includes a shortage of skilled midwives. In 2013, Lira University, a Ugandan Public University, in partnership with Seed Global Health, started the first Bachelor of Science in Midwifery (BScM) in Uganda with a vision to develop a Master of Science in Midwifery (MScM) in the future.

**Objective:**

Evaluate results of Lira University’s Bachelors in Midwifery program to help inform the development of a Masters in Midwifery program, which would expand midwifery competencies in surgical obstetric and newborn care.

**Methods:**

Lira University and Ministry of Health records provided data on curriculum content, student enrollment and internships. The internship reports of the graduate midwives were reviewed to collect data on their employment and scope of practice. Interviews were also conducted with the graduates to confirm the added skills they were able to apply and their outcomes.

**Findings:**

The critical competences incorporated into the Bachelor in Midwifery curriculum included competences to care for pre- and post-operative caesarian section patients or assist in a caesarean section, newborn care (e.g. resuscitation from birth asphyxia), anesthesia, and theatre techniques, among others. Overall, 356 students (40.2% male, 59.8% female) enrolled in the BScM program over the period 2013–2018. Annual data shows an increasing trend in enrollment. Of the 32 graduates in January 2019, 87.6% were employed in maternal and newborn healthcare facilities, and 12.4% were employed in midwifery private practice. Follow-up interviews revealed that the graduate midwives reported positive maternal and newborn outcomes and the ability to practice advanced obstetrics and newborn care skills they acquired from the training.

**Conclusion:**

There is growing interest in a graduate midwifery education program in Uganda for both male and female students. The retention of the graduate midwives in healthcare facilities gives a renewed hope for mothers and newborns, who benefit from their extra obstetrics and newborn care competences in settings where there are neither medical doctors nor obstetricians and gynecologists.

**Recommendations:**

Further, larger tracer studies of the graduate midwives to identify the kinds of obstetric surgeries and newborn care services they ably performed and their corresponding maternal and newborn health outcomes is recommended. Also recommended is advocacy for recognition of extra skills of graduate midwives by health authorities in Uganda and the region.

## Background

### Maternal and Neonatal Mortality

Global maternal mortality ratio (MMR) has remained unacceptably high at 216 per 100,000 live births. It is estimated that 830 women die from pregnancy-related complications around the world every day [1]. In low-income countries, one in 16 women dies of complications from pregnancy. In developed countries, the ratio is markedly lower, at one in 2,800 [2].

Uganda, situated in East Africa, shoulders a high MMR of 336 per 100,000 live births, largely attributable to hemorrhage (34%), hypertension (19%), abortions (9%), sepsis (8%), and indirect causes such as malaria, HIV, and other infections (18%) [3]. Uganda also has a high neonatal mortality rate (27 per 1,000 live births), mostly caused by birth asphyxia and trauma (28.6%), prematurity (27.9%), and sepsis (18.2%) [4].

Proven lifesaving interventions to prevent or treat the causes of maternal and newborn deaths are well known. They include neonatal resuscitation [5], kangaroo mother/father care [6], cord care with chlorhexidine 7.1% [7], antibiotic therapy, helping mother survive bleeding after birth, helping mother survive preeclampsia and eclampsia, and low-dose, high-frequency training in basic emergency obstetrics and newborn care (EMONC) [8], and emergency caesarian section [9].

### Uganda Health System Challenges

In Uganda, as it is in many other low-income settings, the critical challenges affecting the scale up and implementation of the aforementioned lifesaving interventions are shortage of skilled birth attendants; [10] gaps in essential equipment and commodities; [10,11,12] limited hands-on in-service training, mentorship, coaching, and supervision of health workers; [10,11,12] inappropriate training approaches that are theoretical with inadequate simulation and practical sessions; [10] and ineffective engagement of community health workers and leaders to support last-mile distribution of interventions and services [11,12].

**Figure 1:**
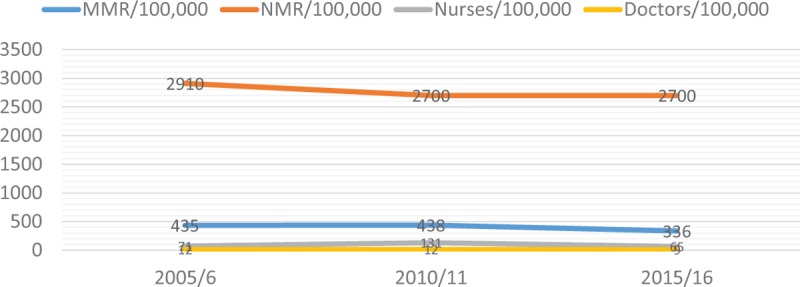
Inverse correlation between health workforce density and maternal and newborn mortality in Uganda (2005–2016).

The shortage of nurses and midwives is of primary concern. The nurse-midwife to patient ratio of 6:100,000 exemplifies the general shortage of health workers in Uganda, which is low compared to World Health Organization (WHO) recommendation of 2.5:1000 [13]. Figure 1 shows how the health workforce density inversely correlates with maternal and neonatal mortality in Uganda from World Health Organization (WHO) Global Health Observatory, WHO Global Atlas, World Bank, UN World Health Statistics, and African Development Bank Group 2005–2016.

### Midwifery Education and Scope of Practice

The high number of births not attended to by skilled birth attendants is partly attributable to the inadequate numbers of nurse-midwives with advanced education in midwifery and maternal neonatal health to address obstetrical and perinatal emergencies.

Notably, the majority of midwives in Uganda are certificate- and diploma-level midwives who are trained to manage normal pregnancy, labor, postpartum care, and newborn care but not the complications of pregnancy, labor, and newborn [10], which are the aforementioned causes of maternal and newborn deaths. Therefore, the problem is a lack of midwives with higher education such as those with Bachelor’s and Master’s degrees to manage complications of pregnancy, labor, postpartum, and newborn health in addition to managing normal pregnancy, labor, and newborn care across the healthcare spectrum. The first-line solution to the above problem is a Bachelor’s-level program in midwifery that imparts the basic nursing skills plus the midwifery specialization required to improve the high maternal and newborn problems in Uganda and the region. The second-line solution is a Master’s level program in midwifery, with specialization in maternal and newborn healthcare.

To justify the need to increase the scope of practice of nurses and midwives with higher education, Dawson et al. [14] demonstrated that obstetric surgical competence and tasks shifting from physicians to non-physicians including nurses and midwives are cost effective, increases access to and availability of maternal and reproductive health services without compromising maternal and newborn health outcomes in Tanzania. Several other studies were conducted in the US and England to establish the effectiveness of nurse-midwives with higher education in the different clinical nursing disciplines (also called advanced practice nurse-midwives). Advanced practice nurse-midwives are shown by various studies in other countries to be very effective in reducing the cost of healthcare and improving general health outcomes of individuals and populations [15,16,17,18,19,20,21,22,23,24,25]. These studies highlighted many positive contributions from the advanced practice nurse-midwives, including improved quality healthcare services and bringing healthcare nearer to the population among others. Overall, the results show that nurse practitioners and advanced practice nurses are most likely to practice in rural settings, low economic areas, and other primary care settings where physicians are scarce. The healthcare services provided by the advanced practice nurses or nurse practitioners are of high quality, low cost, and address the prevalent diseases, health problems, and needs in underserved populations. Related studies conducted on quality of practice and clinical outcomes of nurse practitioners and advanced practice nurses of different specialties found that the nurse practitioners are very relevant and beneficial in primary care, pediatric, maternal, and child health, mental health, geriatrics care, and also in the management of chronic diseases.

Notably, in Uganda as it is in many low-income countries, there is considerable variation in the cadre and the competence of midwives [26]. Unfortunately, not all supposed midwives at a given health facility have the necessary skills to conduct deliveries.

Although midwives with higher education in Uganda, such as those with Master’s degrees, are neither legally termed nor licensed as advanced practice midwives or nurse practitioners as it is in the United States of America (USA) and United Kingdom (UK), their scope of practice is comparable, for example, they can both do independent practice in midwifery. It is therefore plausible to hypothesize that they will address the health needs of Ugandans in comparable quality and a cost-effective way as is being done by advanced practice midwives or nurse practitioners in the US and UK. Currently, there is no data available from Uganda, nor from other settings comparable to Uganda, about the quality and benefits of advancing midwifery education to Bachelor’s or Master’s levels. Nevertheless, we expect the midwives with advanced education to practice at a higher scope than their colleagues with lower education, wherever they will be employed to practice, because the Uganda Nurses and Midwives Council first of all registers and license all midwives with diploma to doctorate levels of education as “registered midwives” without any distinction in their scope of practice. Secondly, Uganda Ministry of Health national health policy [27] defined staffing norms and minimum healthcare package by level of health facility (health centre I–IV, general hospital, regional referral hospital, and national referral hospital), and the definition doesn’t restrict the employment and placement of any midwife with advanced education to work at any level of health facility as long as they qualify to perform the minimum healthcare package recommended to be provided at that level of health facility. Table 1 shows an overview of the Uganda health system, staffing norms, and role of midwives.

**Table 1:** Recommended maternal and newborn health (MNH) services and staffing norms in Uganda by the level of health facility.

Health facility level	Recommended MNH services	Recommended MNH cadres of health workers

National referral hospitals	Provide all maternal and newborn health services that are more comprehensive and advanced than regional referral and general hospitals.	Professors in obstetrics and gynecology, senior consultant obstetricians and gynecologists, consultant obstetrician and gynecologists, Master’s-level midwives and Bachelor’s-level midwives
Regional referral hospitals	Provide elective and emergency cesarean section (C/S) deliveries, laparatomies for ectopic pregnancies, assisted deliveries (vacuum extraction), management of referral of high-risk mothers, management of referral of mothers with severe complications of pregnancy, labor, postpartum, and the newborn, normal deliveries, antenatal care, postnatal care, newborn care, and maternal and child immunizations. Also, provide care to premature babies and asphyxiated newborns in NICU with incubation and CPAP facilities.	Senior consultant obstetrician and gynecologists, consultant obstetrician and gynecologists, Bachelor’s-level doctors, Master’s-level midwives; Bachelor’s-level midwives; diploma midwives; and certificate midwives
District general hospitals	Provide elective and emergency cesarean section (C/S) deliveries, laparatomies for ectopic pregnancies, assisted deliveries (vacuum extraction), management of high-risk mothers, management of complications of pregnancy, labor, postpartum, and the newborn, normal deliveries, antenatal care, postnatal care, newborn care, and maternal and child immunizations	Obstetricians and gynecologists; Bachelor’s-level doctors, Master’s-level midwives; Bachelor’s-level midwives; diploma midwives; and certificate midwives
Health centre IV level primary care facilities	Provide emergency cesarean section (C/S) deliveries, laparatomies for ectopic pregnancies, assisted deliveries, management of complications of pregnancy, labor, postpartum and the newborn, normal deliveries, antenatal care, postnatal care, newborn care, and maternal and child immunizations	Bachelor’s-level doctors; Master’s-level midwives; Bachelor’s-level midwives; diploma midwives; and certificate midwives
Health center III level primary care facilities	Provide health education, antenatal care, both presumptive and laboratory diagnosis and treatment of minor disorders of pregnancy, labor, postpartum and newborn, postnatal care, and maternal and child immunizations	Diploma midwives and certificate midwives
Health centre II level primary health care facilities	Provide health education, antenatal care, presumptive diagnosis, and treatment of minor disorders of pregnancy, postnatal care, and maternal and child immunizations	Certificate midwives
Health Centre I level, also known as village health teams (VHTs)	Provide community-based preventive and promotive services by community health workers such as distribution of information, educational and communication (IEC) materials, door-to-door child immunization, etc.	Volunteer village health teams

Therefore, if the midwives with advanced education are employed in health centre II level, which is an outpatient health post, they will provide health education, antenatal care, maternal care, and childhood care, which is the scope of practice for midwives working in health centre level II. If they are employed and placed at health centre level III, which by design has an additional general ward and maternity ward but lacks a medical doctor, they will be conducting deliveries and management of complications of pregnancy, labor, postpartum, and newborn, plus all the aforementioned maternal and child health services of health centre level II. If they are employed and placed to work in an health centre IV and hospital, which by design has an additional obstetric theatre, anesthesia facilities, and a medical doctor, they will be assisting or actually conducting anesthesia or the cesarian section in addition to the aforementioned maternal and child health services for health centre level II and III. This is expected because medical doctors are in short supply and the few available are busy attending to healthcare needs of other patients, such as those with emergency medicine, internal medicine, pediatrics, surgery, gynecology, orthopedics, ophthalmology, or ear, nose, and throat conditions in addition to general administration and management of the health facility [28]. Arguably, the decentralization plus liberalization of the healthcare service delivery in Uganda to include privately owned health facilities [29] will see that the midwives with higher education will be employed by private sector health facilities because they are less expensive than physicians, and government facilities will continue to hire the less expensive diploma midwives.

## Intervention

As an evidence-based and relevant innovation to address maternal and newborn health problems in Uganda, a four-year Bachelor of Science in Midwifery (BScM) and a two-year Master of Science in Midwifery (MScM) programme (hereafter referred to as graduate midwifery education programmes) were conceived. The scope of practice for midwives that has been used to develop both of the proposed advanced graduate midwifery educational programs is based on the World Health Organization (WHO) and International Confederation of Midwives (ICM) definitions of the midwife. Advanced midwifery practice includes the autonomous provision of healthcare to the girl-child, the adolescent and the adult woman prior to, during, and following pregnancy and post-partum and care of the newborn. This means that the advanced practice midwife gives necessary healthcare, advice, and supervision to women of reproductive health and their children before and during pregnancy, labor, and the postpartum period. The advanced practice midwife provides antenatal care and conducts deliveries on his or her own accord and also provides healthcare to the child, mainly the newborn and infants. The healthcare may include primary healthcare within the community (i.e. basic medical consultations, diagnosis, treatment, health promotion, and preventive interventions such as immunization, growth monitoring, vitamins, and micro-nutrient supplementations); health counseling, information, and education for women, the family, and the community including preparation for parenthood; provision of family planning methods; the detection and treatment of abnormal medical conditions in women of reproductive age, pregnant women, or postpartum mothers and their children; the provision and or referral for specialized care as necessary; medical consultation, medical diagnostic tests, and analysis or surgery; and the execution of primary and secondary interventions for obstetrical emergencies in close collaboration with obstetrician and gynecologists.

The objective of developing graduate midwifery education programs was to incorporate critical surgical obstetrics and newborn care competences and skills required to reduce the leading causes of maternal and newborn deaths in Uganda and sub-Saharan Africa at large. As noted earlier and in Table 2, Uganda’s midwifery problem is not total lack of midwives as there are over 40 health training institutions that churn out over 4,000 [30] of diploma and certificate level midwives per year. Also to note that there are comprehensive training programmes such Bachelor of Science in Nursing (BSc Nursing), Registered Comprehensive Nursing (RCN), and Enrolled Comprehensive Nursing (ECN) that train students in both nursing and midwifery skills and therefore are also regarded as avenues of producing midwives in Uganda.

**Table 2:** Midwifery education system in Uganda.

Training programme	Duration	Award	Entry schemes

MSc Midwifery	2 years	Master’s	Bachelor
BSc Midwifery	4 years	Bachelor’s	UACE/Diploma or Mature age
BSc Nursing	4 years	Bachelor’s	UACE/Diploma or Mature age
BSc Nursing completion	2.5 years	Bachelor’s	Nursing or midwifery diploma
BSc Midwifery completion	2.5 years	Bachelor’s	Midwifery or nursing diploma
RCN	4 years	Diploma	UACE or nursing certificate
Registered Midwifery	3 years	Diploma	UACE or midwifery certificate
RME	1.5 years	Diploma	Certificate in Midwifery
ECN	2.5 years	Certificate	UCE
Enrolled Midwifery	1.5 years	Certificate	UCE

MSc is Master of Science; BSc is Bachelor of Science; UACE is Uganda Advanced Certificate of Education; RCN is Registered Comprehensive Nursing; RME is Registered Midwifery Extension; ECN is Enrolled Comprehensive Nursing; UCE is Uganda Certificate of Education.

The shortage is in midwives with advanced education (Bachelor’s and Master’s level) and thus advanced competence to manage complications of pregnancy, labor, postpartum, and the newborn in addition to managing the normal pregnancy, labor, postpartum, and newborn care in which the enrolled certificate and registered diploma-level midwives obtain training and practice. The duration of training of certificate and diploma-level midwives ranges from 2.5 years for enrolled certificate in midwifery, three years for registered diploma in midwifery and even four years if registered diploma in comprehensive nursing is also included. Comparing the above durations for training enrolled certificate and registered diploma-level midwives and the four years duration for training of the degree level BSc Midwives, the four years is not that long enough to affect interest and enrollment of candidates. In the results section of this paper, we present the attractiveness of the BSc Midwifery programme in terms of trends in student enrolment and graduation rates.

## Methodology

**Figure 2:**
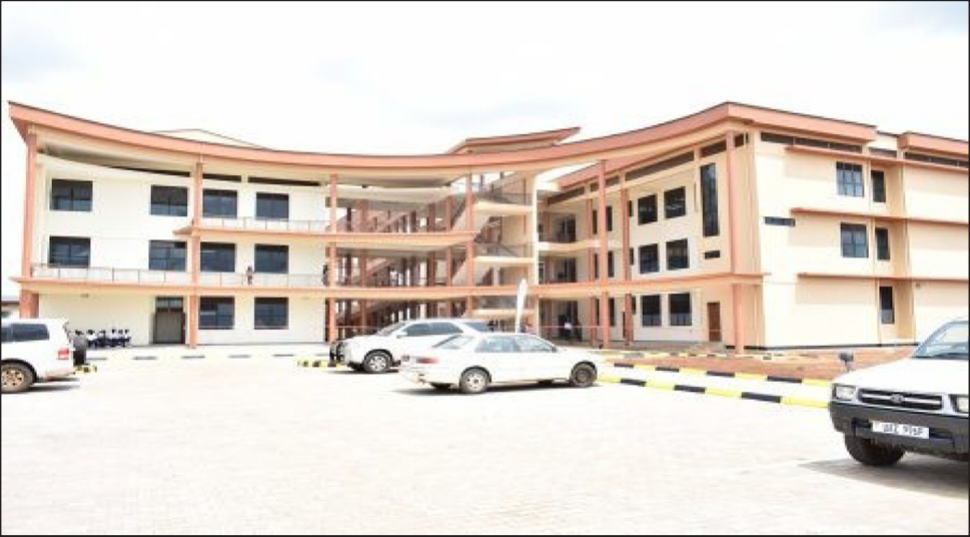
Showing Lira University Teaching Hospital where the Midwifery students conduct clinical practice and patient care.

It was in 2013 that Lira University, a public university in remote Northern Uganda, in partnership with Seed Global Health, implemented the first Bachelor’s degree midwifery education program in Uganda with further possibilities of advancing it to Master’s level by the year 2020. The University has its own Lira University Teaching Hospital (Figure 2).

In addition to the basic midwifery skills such as antenatal care, management of minor maternal and newborn disorders, monitoring of progress of labor, management of normal delivery and newborn care, graduate midwives also have competencies in conducting breach delivery, management of shoulder dystortia, forceps delivery, evacuation and emergency obstetrics and newborn care (EMONC).

Also incorporated are advanced newborn care competences such as neonatal resuscitation, kangaroo mother/father care, cord care with chlorhexidine 7.1%, antibiotic therapy, incubation, phototherapy, Vscan, et cetera. Additional competences include anesthesia and theatre techniques, assisted cesarian section and first and second line management of major maternal and newborn disorders in antepartum, intrapartum, and postpartum periods.

Seed Educators from the USA supported capacity building for Lira University staffs, equipping of skills laboratory, and teaching of students. The Seed Educators were mainly nurse-midwives who came for a yearlong placement. While at Lira University, they contributed to curriculum review, classroom and clinical teaching of students, and faculty and equipping of the clinical skills laboratory with manikins among others.

It is important to note that the BSc Midwifery curriculum has about 60% midwifery and 40% contents of other fields. Other fields include the basic health sciences (i.e. anatomy, physiology, biochemistry, microbiology, pathology, and pharmacology), humanities and behavioral sciences (i.e. sociology and anthropology), public health sciences (i.e. epidemiology and biostatistics, communicable disease control, nutrition, and research methods) and clinical nursing sciences (i.e. primary healthcare, medical, surgical, pediatric, and psychiatric nursing care). These are intentional designed to broaden the student’s foundation and background for better understanding of the midwifery specialty and also for future careers in other fields. Therefore, transition from Bachelor’s to Master’s level in midwifery provides the students with an additional two years for concentrating on midwifery courses with minimal interference from other fields and becoming truly advanced practice midwives. More importantly, it is a period for the students to sharpen competences in managing complications of pregnancy, labor, postpartum, and the newborn, as expected of advance practice midwives. Just like any other health field, the transition from Bachelor’s to Master’s level midwives is likely to attract fewer numbers of midwives who are interested in specializing and becoming future leaders, researchers, and educators of the midwifery profession.

For purposes of understanding the attractiveness of the innovated training in midwifery, Lira University and Ministry of Health records provided data on curriculum content, students’ enrollment, and clinical midwifery internship practice. These were mainly quantitative data on student’s enrollment and completion/graduation disaggregated by year. Similarly, for understanding the relevance, aspects of maternal or newborn outcome and impact of the training, an interactive conversation was held with 22 (14 female, 8 male) of the practicing midwives. Participants’ selection was purposive to ensure balance in the year of completion/graduation from the program, gender, healthcare facility level, public-private ownership, and geographical region location.

The interviews with the selected graduate midwives focused on the advanced midwifery skills they learnt from the program, how successful they were at performing the skills in clinical midwifery practice, and the corresponding maternal or newborn outcomes, whether they are applying them in their workplace, the skills they were not able to practice, and what the challenges or barriers are. The student midwives were contacted through telephone or email. All the interviews with the graduate midwives involved the first author, and they happened during the period July 2019–March 2020. Transcripts of the interviews provided the source of the qualitative data.

Quantitative data from the records such as Lira University student midwives enrollment and graduation numbers were analyzed for percentages and line graphs are used to show trends in enrollment and completion/graduation. Qualitative data from the graduate midwives’ clinical experience interview notes were analyzed to establish whether the graduate midwives are practicing the added skills or not and what the challenges and barriers are in their respective workplaces. Also, simple frequency tallies were used to establish the extent of exposure to a given skill among the graduate midwives. Qualitative data from interviews with the graduate midwives were analyzed using manifest content analysis technique and reported in narrative format with direct quotations.

## Findings

### Internship Placement

**Figure 3:**
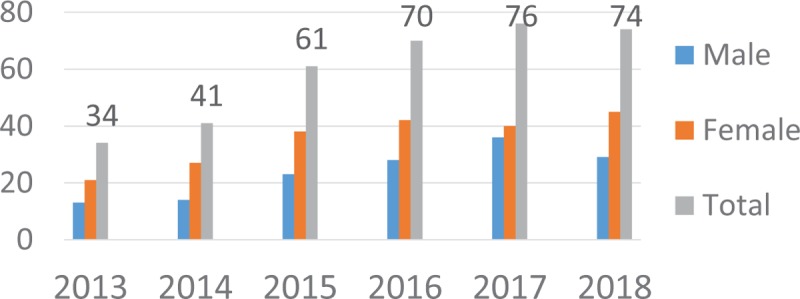
Trend in students’ enrollment into Bachelor of Science in Midwifery programme at Lira University Uganda (2013–2018).

The findings from record review indicated that 356 students (40.2% male, 59.8% female) enrolled in the BSc Midwifery programme over the six years of the program (2013–2018) and the enrollment is on an increasing trend as shown in Figure 3.

The duration of midwifery practice of the graduate midwives ranged from 6–30 months. The graduate midwives with six months of practice experience were those from the 2015 cohort, who started midwifery practice in 2019, while those with 30 months of practice were from among the 2013 cohort, who started practice in 2017. The practice setting spans all the categories and levels of hospitals, a geographical mix, a rural-urban balance, and a public-private balance (Table 3).

**Table 3:** Midwifery practice sites of the graduate midwives.

Practice site	Facility category	Number of midwives

Maracha hospital	Northwestern rural public district general hospital	1
Nebbi hospital	Northwestern rural public district general hospital	1
Lacor hospital	Northern private general hospital	1
Kitgum hospital	Northern rural private general hospital	1
Lira hospital	Northern urban public regional referral hospital	1
Mbale hospital	Eastern urban public regional referral hospital	2
Jinja hospital	Eastern urban public regional referral hospital	2
Mulago hospital	Central urban public national referral hospital	1
Mengo hospital	Central urban private hospital	1
Case hospital	Central urban private general hospital	1
Naguru hospital	Central urban public general hospital	1
Mubende hospital	Central rural public regional referral hospital	1
Bombo hospital	Central rural military general hospital	1
Masaka hospital	Southwestern rural public regional referral hospital	1
Kalisizo hospital	Southwestern public district general hospital	1
Mbarara hospital	Southwestern rural public regional referral hospital	1
Ishaka hospital	Southwestern rural private general hospital	2
Fort portal hospital	Western rural public regional referral hospital	2

### Performance of the advance midwifery skills during internship practice

The findings from the interviews with the graduate midwives shown in Table 4 revealed that all the graduate midwives were able to perform 92% (59 out of the 64) of the advanced obstetrics, gynecology, and abnormal newborn care (neonatology) skills from their training. One of them explained the practice as follows:

During my practice, I was able to carry out the so called “difficult deliveries”, providing care as required even in a resource-limited setting. I was able to conduct so many breech deliveries, twin deliveries, managed emergencies like shoulder dystocia, the mighty postpartum hemorrhage that has claimed lives of many mothers and almost attained 99.9% survival of my mothers and their babies (Female graduate midwife from the 2014 cohort).

**Table 4:** The top 16 advance obstetric and newborn care skills perform by the graduate midwives during clinical practice, their specific roles and the maternal and newborn outcomes.

Sn	Advance obstetric and newborn care skills performed	Tallies total	Tallies by midwives’ specific roles during the performance			Maternal outcome (+ vs. –)	Newborn outcome (+ vs. –)

			Solo actor	Team member	Assisting Physician		

1	PPH management	12	1	10	1	12 vs. 0	NA
2	Pre-eclampsia management	11	11	0	0	11 vs. 0	11 vs 0
3	Breech delivery	10	7	3	0	10 vs. 0	10 vs. 0
4	Neonatal resuscitation	9	8	1	0	NA	7 vs. 2^c^
5	Eclampsia management	8	3	5	0	8 vs. 0	6 vs. 2^a^
6	Twin delivery	8	6	2	0	8 vs. 0	8 vs. 0
7	APH management	7	7	0	0	7 vs. 0	7 vs. 0
8	PROM management	5	3	2	0	5 vs. 0	5 vs. 0
9	Shoulder dystocia management	5	2	3	0	4 vs. 1^b^	5 vs. 0
10	MVA	5	5	0	0	5 vs. 0	NA
11	MRRP	4	4	0	0	4 vs. 0	NA
12	Assisting in C/S delivery	4	0	0	4	4 vs. 0	4 vs. 0
13	D&C	4	4	0	0	4 vs. 0	NA
14	Malaria in pregnancy management	3	3	0	0	3 vs. 0	3 vs. 0
15	UTI in pregnancy management	3	3	0	0	3 vs. 0	3 vs. 0
16	Premature baby care	3	0	3	0	NA	2 vs. 1

^a^ One baby was a fresh still birth and the other died from the from neonatal intensive care unit (NICU). ^b^ One mother got second-degree tear, which was repaired. ^c^ One baby died on the resuscitation table and the second one died in the NICU.

As shown in Table 4, there were 16 top advanced obstetrics and newborn care skills performed by most of the graduate midwives at their clinical workplaces. These include postpartum hemorrhage (PPH) management, pre-eclampsia management, breech delivery, neonatal resuscitation, eclampsia management, twin delivery, antepartum hemorrhage (APH) management, premature rupture of membranes (PROM) management, shoulder dystocia management, manual vacuum aspiration (MVA) of retained products of conception, manual removal of retained placenta (MRRP), assisting in cesarean section (C/S), dilatation and curettage (D&C), urinary tract infections (UTI) in pregnancy management, and premature baby care.

Many of the graduate midwives placed in rural district general hospitals where there are no obstetricians and gynecologists performed most of the added obstetrical skills solo. One of them explained his solo management of a case of premature rupture of membrane as follows:

I received a prime gravida with complaint of draining liquor, I observed her and she was not pushing or grunting. The abdomen had no previous cesarian section scar, no horizontal ridges across lower abdomen. I felt the abdomen for contractions frequency; duration was mild, each lasting less than 20 seconds; fundal height was 38/40; fetal lie was longitudinal; presentation was cephalic and fetal movement not felt. I listened to the fetal heartbeat, was 140 beats/minute. I checked for maternal blood pressure (BP), temperature, pallor, sunken eyes, all were normal. On vaginal examination, there was no bulging perineum, no vaginal bleeding, but there was visible leaking amniotic fluid, which was not meconium stained. I performed digital vaginal examination and found cervical dilatation was 2 cm and < 2 contraction in 10 minutes. I administered prophylaxis antibiotics Ampicillin 2g start. Did and obtained Bishop Score of 8. I chose to perform induction of labor with hormonal method using Oxytocin. I administered Oxytocin IV 2.5 IU in 500 mL of normal saline at start infusion rate of 10 drops/minute. I increased infusion rate by 10 drops every 30 minutes (max 60 minutes) until good contraction pattern was established (3–5 contractions in 10 minutes, each lasting >40 seconds). I maintained this rate until delivery was complete. The mother had normal spontaneous vaginal delivery to baby girl, 2.5 kg. Both mother and baby were discharged in good conditions (Male graduate midwife of 2014 cohort).

Additionally, there are other added skills performed by at least two of the midwives in their respective clinical practices with positive outcomes for both the mothers and babies. These include assisting in laparotomies for ectopic pregnancies, cord prolapse delivery, cutting and repairing of episiotomies, repairing of second-degree tears, abortion management, cervical cancer screening, prolonged labor management, uterine rupture management where one mother died, post-abortion care, obstructed labor management, induction of labor, vacuum extraction, preoperative care, postoperative care, prolonged labor management and neonatal sepsis management. One of the graduate midwives explained how he successfully managed postpartum hemorrhage and how the success brought him joy as a midwife:

PPH (postpartum hemorrhage) claims many lives of mothers in Uganda, and at times it poses a nightmare for midwives and doctors. During my three-month rotation in labor suite, I faced eight different PPH cases. Terrifying at the first encounter but with more composure, I resolved and determined my mindset and well prepared with equipment and necessary materials, I managed PPH cases after personally conducting the deliveries or after the student midwives (certificate and diploma midwife) with help of mainly senior midwives and intern doctors. This was through various interventions, which included uterine massage, Oxytocin infusion, expelling clots, emptying the bladder by urinary catheter, repairing mainly second-degree tears, and using balloon tamponade. It brought much joy and satisfaction to me when homeostasis was achieved and the mother was in stable condition with healthy baby (Male graduate midwife of 2014 cohort).

More so, there are other added skills performed by at least one graduate midwife during their clinical practices with positive outcomes for both the mothers and babies. These include severe hyperemesis gravidarum management, assisting in laparotomies for ectopic pregnancies, family planning method particularly intrauterine contraceptive device and implants counseling and insertion, birth asphyxia management, fetal distress management, intrauterine fetal death management, sickle cell crisis in pregnancy management, intrauterine fetal resuscitation, infertility management, cervical dystocia management, post-term pregnancy management, birth defect counseling and referral, compound presentation management, arm prolapse management, placenta pacrater management, triplet delivery, sexually transmitted infection (STI) treatment, molar pregnancy management, unconscious mother in labor management, ectopic pregnancy management, neonatal jaundice management, anemia in pregnancy management, puerperal sepsis management, neonatal necrotizing enterocolitis management, neonatal hypothermia management, and neonatal respiratory distress management.

Another most exciting bit was when I was managing third stage of labor after I delivered the baby. I followed all the steps of AMTSL (active management of third stage of labor), but to my surprise, the placenta could not be delivered for some good time despite of all the intervention. Mother was not bleeding and the placenta showed me that it was not about to be delivered. I thought of option B that was manual removal. I shared the option with my senior; she said its fine go on. I prepared to deliver the placenta manually, guess what, when held the cord with my left hand and as I followed the cord with the other hand trying to locate the edges of the placenta, I realized the cord was entering to the uterine wall. I was shocked. We called the gynecologist; he told me it was placenta pacrater. She was later taken in for hysterectomy (Female graduate midwife from 2014 cohort).

There were only five added skills that the graduate midwives were not able to perform during their clinical practice. These were performing cesarean section, laparotomies for ectopic pregnancy, symphysiotomy, and repair of third- and fourth-degree perineal tears. The challenges and barriers are listed in Table 5, which ranges from few or total lack of patients with the indications for the intervention. Also, some graduate midwives were denied the chance to perform the procedures by their supervisors due to the misperception that they are not qualified enough or the procedure or intervention should be performed by physicians who have the authentic license or permit to perform the procedures.

**Table 5:** Challenges and barriers for the graduate midwives’ failure to perform some of the added skills.

Sn	Added skills not or underperformed	The challenges or barriers responsible

1	Delivering babies with shoulder dystocia from both rural and urban regional referral hospitals	Cases are rare, high competition amongst health workers for the few available cases; some clinical supervisors prefer to refer the cases to theatre for operation by physicians instead of first giving the chance for the graduate midwives to manage.
2	Performing symphysiotomy from urban and rural regional referral hospitals and even rural district general hospital	Clinical supervisors block the graduate midwives from performing symphysiotomy, on the premise that they are not experienced enough to safely perform the procedure
3	Performing of C/S delivery from urban and rural regional referral hospitals and even rural district general hospitals	Clinical supervisors block the graduate midwives from performing C/S delivery, on the premise that they are not licensed for the role
4	Performing laparotomies for ectopic pregnancies from urban and rural regional referral hospitals and even rural district general hospitals	Clinical supervisors block the graduate midwives from performing C/S delivery, on the premise that they are not licensed for the role
5	Repairing of third- and fourth-degree perineal tears from national and regional referral hospitals	Clinical Supervisors block the graduate midwives from repairing of third and fourth degree perineal tears, on the premise that they are not licensed for the role

It is important to note that even for skills like manual vacuum aspiration, dilatation and curettage, vacuum extraction, forceps deliveries and assisting in cesarian section (C/S) deliveries, which were performed by a few of the graduate midwives from rural hospitals, it is not universal as such. Some clinical supervisors in some of the above hospitals blocked the graduate midwives from performing the procedures on the ground being that they are not qualified enough, or the government of Uganda has not yet licensed them to perform those skills. Other graduate midwives simply did not perform the added skills because of lack of cases with the indications for the intervention or just lack of equipment or facilities. Several graduate midwives commented on this:

I was not permitted to perform cesarean section, exploratory laparotomy for ectopic pregnancy, nor vacuum delivery. Most reasons pointed towards lack of clear guidelines and policies in relation to midwives’ license to perform such procedures (Male graduate midwife from 2013 cohort).

Some conditions like shoulder dystocia are very rare. I haven’t come across one in six months. Here, procedures like manual vacuum aspiration, dilatation, and curettage are purely for doctors (Female graduate midwife from 2014 cohort).

I did not do cases concerning shoulder dystocia, deliveries by vacuum extraction, manual removal of the retained placenta because there was high competition for skills among the intern midwives, intern doctors, and students (Female graduate midwife from 2014 cohort).

### Post-internship Work and Application of the Added Skills

Of the 34 students in the first 2013 cohort, 94.1% graduated and completed internships in record time. Post-clinical midwifery internship employment status of the 32 midwives who completed internships show that by January 2019, 25% were retained at Lira University Hospital as Midwives and Teaching Assistants, 62.5% gained employment in various maternal and newborn healthcare programs across Uganda. The remaining 12.4% ventured into private midwifery practice. Overall, there is 88% retention of the graduates at in-country hospitals.

As noted above, quite a good number of the graduate midwives from the 2013 cohort were retained at Lira University and Lira University Teaching Hospital. In the teaching hospital, they are involved in teaching students, supervising and mentoring diploma midwives, and research in addition to participating in managing mothers with obstetrics and gynecological conditions. The work of the graduate midwives in teaching hospitals is explained as follows:

I am currently working at Lira University and Lira University Teaching Hospital as a Teaching Assistant in the Department of Nursing and Midwifery. I teach, set examination, and supervise students in the clinical areas, attend ward rounds, give treatment to patients, monitor mothers in labor, conduct deliveries, supervise other clinical staffs [diploma midwives] in my unit and participate in research. I am able to apply most of the obstetrics skills l learnt like managing a mother with pre-eclampsia and eclampsia, PPH (post-partum hemorrhage), shoulder dystocia, resuscitating newborns, manual removal of retained placenta, etc. (Female graduate midwife from 2013 cohort).

Notably, the graduate midwives from the 2014 cohort have also finished internship practice and entered into the job market. Of the nine of them in this study sample, seven are employed and working. Two of them were employed in Sanyu Africa Research Institute – one as a research midwife, where she is applying research skills – and one in the International Rescue Committee as a reproductive health officer, responsible for programming reproductive health services in a refugee settlement. Some of them were employed as nursing officers in midwifery at Mbarara Regional Referral Hospital, which has all the necessary equipment or instruments for managing obstetric complications and therefore are able to apply the added obstetrics skills. One of them described the following:

I am currently working with Global Health Collaboration that partners with Mbarara Regional Referral Hospital as a nursing officer–midwifery, and l do clinical work in the obstetrics and gynecology department. I am able to apply the following abnormal midwifery skill: management of PPH, breach delivery, episiotomy and repair of both episiotomy and perineal tears up to second degree, newborn resuscitation, twin deliveries, and cord prolapse deliveries (Male graduate midwife from 2014 cohort).

Some graduate midwives from the 2014 cohort are employed and working in remote lower-level public health centres (Warr health centre IV in Zombo district in northern Uganda and Namisindwa health centre III in Namisindwa district in Eastern Uganda). The one working in health centre IV with a theatre facility is able to assist in cesarian section delivery, conduct pre- and post-operative care and neonatal resuscitation. The one working in health centre III with a maternity ward but no theatre and equipment for managing obstetric cases is only able to conduct the routine antenatal care, delivery of mothers with normal labor, referral of high-risk mothers to higher-level health facilities and managing the maternity ward. The work of the graduate midwives in the lower level primary care health facilities with maternity ward but without theatre or equipment for managing obstetric cases is described as follows:

I work with Namisindwa local government, which is one of the newly created districts in Uganda, as nursing officer – midwifery in health center three. It is a very hard-to-reach area with a health center three being the highest level of care facility in the entire district.

The work I do are: organizing and participating in departmental meeting; ordering, balancing, and keeping drugs and supplies records updated; making of duty rosters and ensuring all duties are covered accordingly; writing weekly and monthly reports; coaching and mentoring the junior staff on new midwifery knowledge and skills to enable efficient and quality service or care delivery to all mothers and their unborn babies; working hand-in-hand with visiting medical personnel to improve on services given to clients; [and] performing activities like ensuring cleanliness of the department, managing deliveries, providing general nursing care to all clients entrusted in my care, preventing and treating minor ailments, early identification of complications and referring for appropriate care to Mbale regional referral hospital because the district has no capacity to handle any emergency cases (Female graduate midwife from 2013 cohort).

The practice is a bit different for those employed in urban private general hospitals. There are many high-risk mothers but their obstetricians and gynecologists are always available for them and do much caring to prevent the occurrence of the obstetric complications. Consequently, the graduate midwives conduct normal deliveries, normal newborn care and postnatal care where there are limited chances of applying their added obstetrics skills. One of them had this to explain:

Currently, I am working at Case Hospital as a midwife for experience as I am looking for a better job. I am majorly working in labor ward and NICU (Neonatal Intensive Care Unit). Being a private setting [hospital], it is a different protocol all together. Mostly, we work hand-in-hand with the gynecologists and pediatricians. They are there for each of their mothers and newborns. In most cases, they do a lot of preventive measures as a result there are few cases of abnormal midwifery. The few are pre-eclampsia, twin delivery, PROM (premature rupture of membranes), etc. But personally the only challenge I faced was during PPH (post-partum hemorrhage) caused by cervical tear (Female graduate midwife from 2014 cohort).

## Discussion

Our study showed increasing trends in both enrolment and graduation from the four-year-long training for graduate midwives in Uganda. This is an evidence that the training is both attractive and attainable. The four years of the training is worth the extra obstetric surgical skills that the students acquire if compared to the four years training for diploma in comprehensive nursing, the three years training for diploma in midwifery and 2.5 years training for certificate in midwifery where the candidates come out with skills for providing normal midwifery and newborn care.

The above finding from Uganda concurs with finding of career retention survey conducted among Bachelor’s and Associate’s degree nurses in the US state of Vermont that found that Bachelor’s nurses were employed for longer periods of time, despite Bachelor’s education requiring more time to complete (4 years) compared to Associate’s Degree nurses (2–3 years) [31]. In the time of health workforce shortage, the longer duration of careers for Bachelor’s nurses compared to Associate’s Degree nurses is an important social return.

Our study further revealed that the graduate midwives are retained to work in primary maternal and newborn care facilities across Uganda. This is not a mere point on the relevance of the training but also gives a renewed hope of mothers and newborns benefiting from their added obstetric surgical competences and also providing leadership to certificate and diploma midwives in primary care facilities. The employment of the graduate midwives in primary care facilities like hospitals and health centres ensures quality healthcare are nearer to where pregnant mothers and their newborns live. More so, our study revealed that graduate midwives are employable in academic facilities where they provide teaching and mentorship to students, certificate and diploma level midwives in both clinical and classroom areas. Also exciting to note is our finding that graduate midwives are employable in research roles, where they participate in advancing the midwifery profession with new knowledge.

The aforementioned employment opportunities for graduate midwifery educational programmes are not surprises because the training equips students with clinical, research, management, leadership, and teaching skills to practice in the primary care settings (i.e. health centres), in hospitals, schools of nursing and midwifery, universities, research organizations and other settings including community-based healthcare programming. With the increasing awareness amongst the job market stakeholders about the added obstetrical surgical skills of the graduate midwives, the employment prospects for the graduate midwives are becoming broader than the ones identified from this study, to span governmental [32] and non-governmental organizations and the private sectors as well in neighboring countries like Kenya [33], as listed in Table 6.

**Table 6:** Employment Prospects for Graduate Midwives in Uganda.


Primary healthcare providers in midwifery and maternal child health specialties
Midwifery clinical specialists
Clinical researcher, research coordinators, quality control and monitoring officers
Midwife educators or lecturers
Principal nursing officers – midwifery in governmental, non-governmental, and private healthcare sectors
Reproductive health, maternal child health program/project officers or managers
Clinical leaders in reproductive health and family planning service organizations
Private midwifery practice in homes or maternity homes
Primary healthcare providers in midwifery and maternal child health specialties
Entrepreneurs providing midwifery/maternity services, primary, and reproductive health services


The above finding concurs with the findings from the survey conducted among Bachelor’s and Associate’s Degree nurses in the US state of Vermont, which indicated that Bachelor’s nurses enter careers earlier and are employed for longer durations compared to the Associate’s degree nurses [31]. In the time of a health workforce shortage, the time to career is an important social return, indicating that there were more job opportunities for Bachelor’s nurses, enabling them to get jobs more quickly compared to their Associate’s degree counterparts.

Importantly, our study revealed that the graduate midwives are helping to bridge obstetric surgical skill gaps in primary care facilities in Uganda where there are neither medical officers nor obstetricians and gynecologist physicians or are available but busy with provision of other medical services or administrative duties. This to us is the most critical contribution where the graduate midwives will register future impact on maternal and newborn health outcomes.

Our data revealed that while performing all the roles and responsibilities, the graduate midwife uses evidence-based guidelines, protocols, and approaches from midwifery, nursing, medical, and other health sciences. The graduate midwives are trained to be able to take leadership and assume responsibility for providing appropriate healthcare services including the prescribing, administering and dispensing of pharmacologic agents including drugs and therapeutics especially in the antenatal, labor, and postnatal care areas. Notably, in the Uganda health system, even the certificate and diploma midwives are already licensed by health authorities to prescribe, administer, and dispense drugs for treatment of minor disorders in pregnancy, labor, and postpartum, including iron sulphate, folic acid, and anthelminthic drugs, including those for prevention of malaria in pregnancy. They are, however, restricted from treating high-risk mothers, such as those with underlying medical conditions (diabetes, sickle cell disease, heart disease, etc.), whom they are expected to refer to physicians, particularly obstetricians and gynecologists. The certificate and diploma midwives are also restricted from managing mothers and newborns with severe complications of pregnancy, labor, and postpartum such as those with hemorrhage, pre-eclampisa, etc. The restriction is based on the fact that their short 2.5–3 years training does not include skills for managing high-risk mothers and those with severe complications of pregnancy, labor and postpartum. Despite the aforementioned restrictions of certificate and diploma midwives based on their limited competence, Uganda hasn’t yet managed to train enough physicians to deploy in all levels of primary care facilities. Health centres level II–III, and even many level IV primary care facilities in Uganda, do not have physicians to manage the aforementioned maternal and newborn high-risk conditions and complications. Therefore, the expanded skills and scope of practice of the graduate midwives will add values to every level of Ugandan health system, as explained in Table 7.

**Table 7:** Examples of value addition from graduate midwives to the Uganda health system.

Ugandan health system	Value additions from the graduate midwives well and above those being provided by the existing certificate and diploma midwives

Health centre level I, which is a mobile voluntary village health team without physical infrastructure.	No value addition
Health centre level II primary care facility, which has an outpatient department without maternity ward or physician	Increased management of moderate to severe complications of pregnancy, postpartum, and the newborn. This is because the certificate and diploma midwives can only manage the minor disorders. Additionally, there will be reduction in referrals to higher-level facilities of mothers and newborns for management of moderate-severe complications and thus saving time and cost to families. Lastly, there will be an improved leadership and management in the maternal child health department.
Health centre level III primary care facility which has an outpatient department, maternity ward, laboratory testing but no theatre or physician	Increased management of moderate to severe complications of pregnancy, labor, postpartum, and the newborn. This is because the certificate and diploma midwives can only manage the minor disorders. More so, there will also be reduction in presumptive diagnosis of maternal and newborn conditions from increased ordering of laboratory testing from the graduate midwives, which will improve the accuracy of diagnosis, treatments, and reduce drug wastage. Additionally, there will be reduction in referrals to higher-level facilities of mothers and newborns for management of moderate to severe complications including complications of labor and thus saving time and cost to families. Lastly, there will be an improved leadership and management in the maternal child health department, labor, and postnatal wards.
Health centre level IV primary care facility which has an outpatient department, maternity ward, laboratory testing, theatre and physician. The physician often one position is also administrative and management duties of the health centre. In addition, to note that most of the level IV facilities have no physicians nor anesthetic officers for the operation of the theatre.	Increased management of moderate to severe complications of pregnancy, labor, postpartum and the newborn. This is because the certificate and diploma midwives can only manage the minor disorders. More so, there will also be reduction in presumptive diagnosis of maternal and newborn conditions from increased ordering of laboratory testing from the graduate midwives, which will improve the accuracy of diagnosis, treatments and reduce drug wastage. Additionally, there will be reduction in referrals to hospitals of mothers and newborns for management of moderate to severe complications including complications of labor and thus saving time and cost to families. Also, there will be an improved leadership and management in the maternal child health department, labor, and postnatal wards. There will be increased cesarean section rate and improved outcomes of mothers from theatre as the graduate midwives will improve preoperative care, increase assistance of the physician during cesarean section, improve anesthesia and theatre techniques, improve postoperative care, and improve newborn care from resuscitation. If the facility does not have a physician, the graduate midwives will perform emergency cesarean section if licensed to do so.
Hospitals	Increased management of moderate to severe complications of pregnancy, labor, postpartum, and the newborn. This is because the certificate and diploma midwives can only manage the minor disorders. More so, there will also be reduction in presumptive diagnosis of maternal and newborn conditions from increased ordering of laboratory testing from the graduate midwives, which will improve the accuracy of diagnosis and treatments and reduce drug waste. Additionally, there will be a reduction in referrals to hospitals of mothers and newborns for management of moderate to severe complications, including complications of labor and thus saving time and cost to families. Also, there will be an improved leadership and management in the maternal child health department, labor, and postnatal wards. There will be increased cesarean section rate and improved outcomes of mothers from theatre as the graduate midwives will improve preoperative care, increase assistance of the physician during cesarean section, improve anesthesia and theatre techniques, improve postoperative care, and improve newborn care from resuscitation. If the facility does not have a physician, the graduate midwives will perform emergency cesarean section if licensed to do so.
District health office as assistant district health officer – nursing and maternal child health	Improvement in the management, particularly midwifery, maternal, and child health services in the district from quality technical monitoring, supervision, mentorship of certificate and diploma midwives at health facilities.
Health development partners working on maternal child health programs as program officers, technical advisors, project managers, etc.	Improvements in quality of programs for midwifery, maternal, and child health in the district health office and health facilities.

With increasing awareness of the value additions from the graduate midwives’ added obstetric skills, we listed in Table 7 all the practice settings within Uganda health system where the graduate midwives would qualify to seek employment should there be job openings in midwifery, maternal, and child health fields.

The finding of our study concurs with several previous studies that pointed to a strong link to improvement in patient outcomes from four-year bachelor’s level education of nurses in the Europe. Aiken et al. (2014) in a study in nine European countries found that every 10% increase in a bachelor’s degree nurse was associated with a 7% decrease in likelihood of inpatient dying [34].

Our data found out that the major post-training practice challenge experienced by the graduate midwives is lack of recognition of their extra obstetric surgery skills by other health cadres and thus denying them official opportunities to use the added obstetric surgical skills to save lives. Another challenge was the deployment of the graduate midwives in lower-level primary care facilities, such as health centre III which does not have the necessary capacity (in terms of infrastructure, equipment, protocols, supplies and drugs) to allow the graduate midwives apply the obstetric surgical skills.

The above findings concur with a study conducted in Malawi, which indicated a tension between Bachelor’s degree nurses and diploma nurses in a clinical setting, whereby the diploma nurses often do not feel confident or want to support the degree nurses [35]. Tension between cadres is normal during introduction of new cadre of health professionals. It should be prevented through a scheme of service with updates to cater for scope of practice of the new cadre. Uganda has changed its scheme of service to include Bachelor’s and Master’s nurses and midwives [36] and this change will prevent tension between various cadres of nurses and midwives.

## Conclusion

Graduate midwifery education at Lira University in Uganda is an innovation that is attracting high student enrolment. The retention of the graduates into clinical practice settings in Uganda further provides opportunities for addressing maternal and newborn health problems, quality issues in midwifery practice, gaps in clinical research, and gaps in leadership of the midwifery profession in Uganda.

## Recommendations

Larger tracer studies of the graduate midwives to identify the kinds of obstetric surgeries and newborn care services they ably performed and their corresponding maternal and newborn health outcomes is recommended. Also recommended is advocacy for recognition of extra skills of graduate midwives by the health authorities in Uganda.
